# Microbial community structure is affected by phage-resistance associated increases in host density

**DOI:** 10.1093/femsec/fiaf027

**Published:** 2025-03-18

**Authors:** Meaghan Castledine, Daniel Padfield, Rai Lewis, Angus Buckling

**Affiliations:** College of Life and Environmental Sciences, Environment and Sustainability Institute, University of Exeter, Penryn, Cornwall TR10 9EZ, United Kingdom; College of Life and Environmental Sciences, Environment and Sustainability Institute, University of Exeter, Penryn, Cornwall TR10 9EZ, United Kingdom; College of Life and Environmental Sciences, Environment and Sustainability Institute, University of Exeter, Penryn, Cornwall TR10 9EZ, United Kingdom; College of Life and Environmental Sciences, Environment and Sustainability Institute, University of Exeter, Penryn, Cornwall TR10 9EZ, United Kingdom

**Keywords:** community ecology, bacteriophage, phage resistance, evolutionary ecology

## Abstract

Lytic bacteriophages (‘phages’) can limit bacterial densities and shape community structure, either directly through lysis or indirectly through costs to resistance. However, phages have also been reported to have no, and in some cases even positive, effects on host densities. Here, we investigate the mechanisms behind an increase in host density in *Variovorax* sp. populations following a fixation of resistance that was maintained after phage extinction. Our results demonstrate that the density increase was a genetic trait coinciding with resistance emergence. Growth curves showed that phage resistance shifted population growth curves such that density was higher in the death phase. This density-increasing effect of resistance had important implications for community structure with phage-resistant *Variovorax* decreasing the density of a conspecific. That resistance to lytic phage can increase host densities has implications for wider ecology and phage therapy, where lytic phages are presumed to have negative effects on their hosts.

## Teaser

Microbial community structure is affected by phage resistance evolution in *Variovorax*, which increases host density. Created in BioRender. Castledine, M. (2025) https://BioRender.com/q92i397

## Introduction

Bacteriophages are hypothesized to have major impacts on the structure of microbial communities, and in regulation of wider nutrient cycles (Winter et al. 2010, Breitbart 2012, Breitbart et al. 2018). Part of this assumption comes from the ubiquity of bacteriophages (Clokie et al. 2011). By lysing their hosts, phages can suppress fast-growing bacteria densities and give a relative advantage to slow-growing or rarer microbial taxa (Winter et al. 2010). Phages are shown to lyse algal blooms, resulting in crashes in algae abundances and nutrient release, which benefits the surrounding community (Breitbart et al. 2018, Flynn et al. 2022, Liao et al. 2023). Furthermore, a common assumption of many models is that phage resistance is costly (Winter et al. 2010), which leads to suppression of dominant or previously fast growing bacteria populations (Gandon et al. 2008, Winter et al. 2010).

However, the effects of phage resistance on host phenotypes, and the associated cost, is highly system specific. If evolving phage resistance typically involves modification of a receptor essential to host growth, resistance is likely to be associated with a growth rate cost (van Houte et al. 2016). For example, to become resistant to OMKO1, *Pseudomonas aeruginosa* mutates an efflux pump required for drug resistance (Chan et al. 2016). As a result, while antibiotics are present, resistance to OMKO1 imposes a cost to *P. aeruginosa* (Chan et al. 2016). Similarly, phage resistance involving mutations to flagella or pili are further shown to slow bacteria growth rates (Buckling et al. 2006, Li et al. 2022). However, there are numerous examples of phage resistance being noncostly to growth including in *Pseudomonas syringae* (Koskella et al. 2012), *Synechococcus* (Lennon et al. 2007), and *P. aeruginosa* (Castledine et al. 2022).

Phage resistance may also indirectly increase host fitness (beyond the direct effect of resisting phage). For instance, increased biofilm production is a phage resistance mechanism, which physically blocks phage access to bacteria cells (Salathé and Soyer 2008, Harper et al. 2014, Abedon 2023). Biofilm production has consequential fitness benefits in resisting wider environmental stress such as antibiotics (Crabbé et al. 2019), and can be advantageous in competition (Oliveira et al. 2015). Phages can also accelerate the molecular evolution of their hosts and select for hypermutator strains, therefore giving increased adaptive potential to secondary stressors (Pal et al. 2007, Jayaraman 2011). *Pseudomonas fluorescens* phage has been shown to increase host densities in soil, although only when grown in monoculture (in the absence of a soil community) (Gómez and Buckling 2011). Here, phages are hypothesized to have increased bacterial densities by reducing intraspecific competition through slowing of growth rates or facilitating diversification into separate ecotypes (Gómez and Buckling 2011), although the exact mechanism is unknown.

Understanding how phages can increase bacteria densities is important for phage therapy, where increasing bacteria densities would be undesirable (Abedon 2023). Furthermore, investigating this effect has important implications for our understanding of how phages shape microbial communities where, in nature, phages are presumed to have predominantly negative effects on their host densities (1, 3). In a previous study, we noted that densities of *Variovorax* sp. increased following phage exposure and fixation of phage resistance (Castledine et al. 2024a) even when phage had been driven extinct. Here, we investigate the mechanism underpinning phage-mediated increases in *Variovorax* densities, and the consequence this has for the composition of a three species synthetic bacteria community. This microbial community is composed of soil bacteria, which stably coexist, an important aspect of natural communities (Castledine et al. 2024b). Therefore, this allows us to test how phage affect community structure of species, which are likely to interact in nature. We show that phage resistance is associated with density increases resulting from a shift in growth curves that led to higher densities in the death-phase. Phage resistance had ramifications for community structure with decreases in the proportion of another community member which is parasitized by *Variovorax*.

## Materials and methods

### Experimental evolution

**Figure 1.**
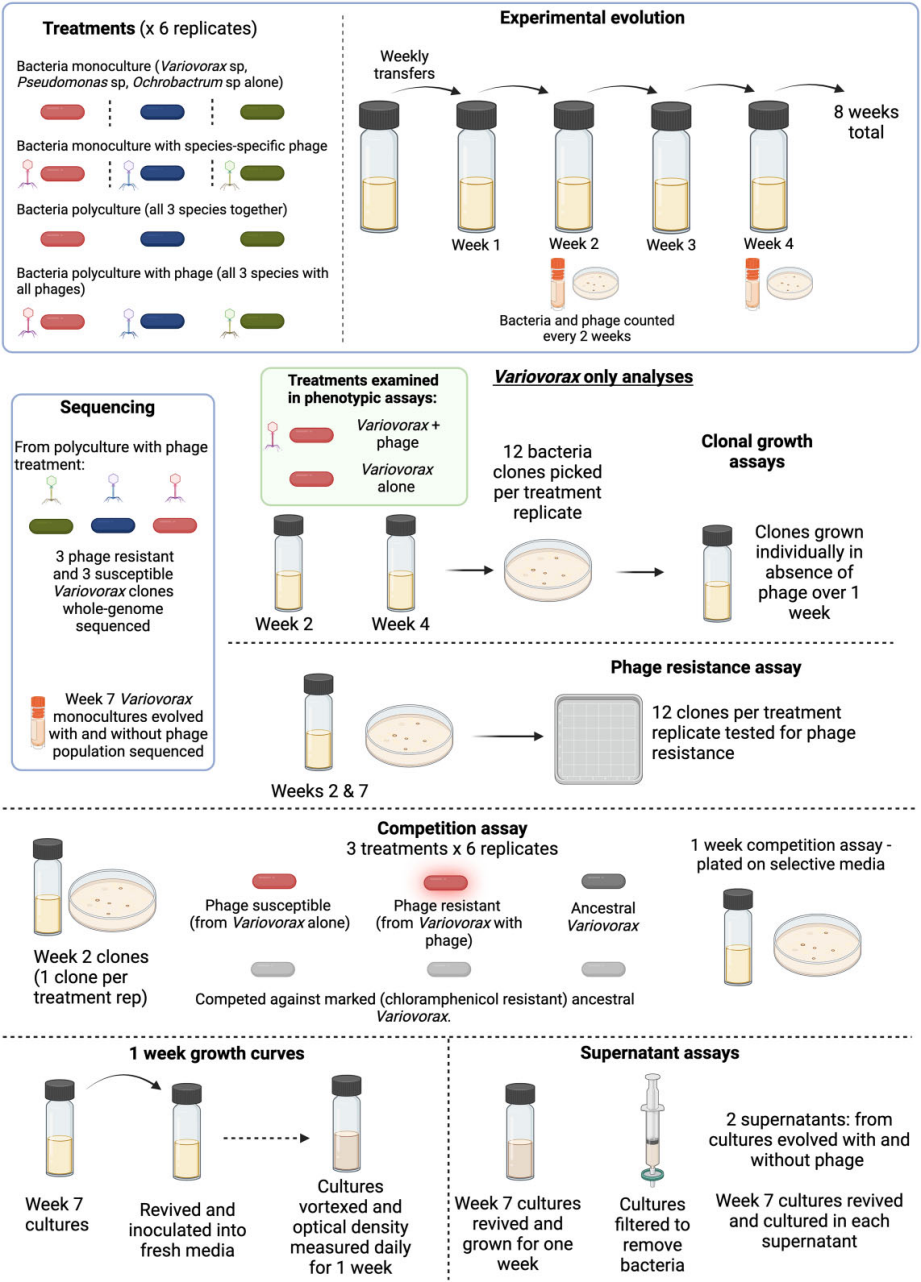
Overview of the experimental design in which *Variovorax* and its phage evolved in monoculture and polyculture, and assays performed to understand the mechanism of this density increase. Sequencing analyses and phenotypic assays aimed to determine the mechanism by which phages led to increased *Variovorax* densities across experimental evolution. Created in BioRender. (Castledine 2024) BioRender.com/c73o264.

*Variovorax* sp. is a common soil bacterium with antiphage defences including Zorya Type III, ietAS, dXTPase, DRT (defence-associated reverse transcriptase), retron type XIII, DNA-modification systems and restriction-modification type II (Supplementary Data, identified using PADLOC; Payne et al. 2022). In a previous study, species *Ochrobactrum* sp., *Pseudomonas* sp., and *Variovorax* sp. were grown as monocultures and as a three-species polyculture with and without their respective phage (*Ochrobactrum* phage ORM_20, *Pseudomonas* phage CH7FMC, *Variovorax* phage VAC_51) (Castledine et al. 2024a). Experimental treatments included: monoculture, monoculture with phage (each bacterial species individually with only its species-specific bacteriophage), polyculture (all three bacteria present), and polyculture with phage (all three bacteria with all three phages present) (Fig. 1). Each treatment was replicated six times. Experimental set-up is described in Castledine et al. (2024a). Briefly, cultures were established using isogenic strains of each bacteria and phage, with phage added at an multiplicity of infection (MOI) of 0.01 (to phage present treatments). Cultures were grown in 1/64 tryptic soy broth (TSB; diluted in demineralized H_2_O), at 28°C and transferred weekly to fresh medium. Bacteria and phage densities were estimated every 2 weeks. For *Variovorax* cultures, we estimated phage resistance at weeks 2 and 8 in monoculture evolution lines (phage present and absent). Phage resistance was analysed using spot assays with ancestral phage.

### Clonal density measures

We first wanted to assess whether higher densities conferred to phage-exposed populations was a heritable trait observable in single clones picked and regrown in the absence of phage. From week 2 and 4 cultures (the weeks preceding and following higher densities), 12 colonies were isolated from each replicate from monoculture treatments. Colonies were individually grown in 1/64 TSB and density normalized as described previously (Castledine et al. 2024b). To fresh 6 ml 1/64 TSB, ∼10^6^ colony forming units (CFUs) of each clone were added to individual vials. Cultures were grown for 1 week, frozen, and plated from frozen onto KB agar as previous (Castledine et al. 2024a). Colonies were counted from agar plates to estimate bacteria density after 2 days at 28°C.

### Clonal and population sequencing and bioinformatics

To gain insight into the mechanism of resistance and whether this could be linked to changes in bacterial density and growth rate, isolates were sent for whole genome sequencing (WGS). Three phage-resistant and three phage-susceptible clones from three independent week 2 polyculture lines were selected for WGS. Polyculture lines were selected as these were the only treatment, which had phage-susceptible and -resistant clones coexisting (100% resistance was found in monoculture lines). This allowed for direct comparison between different clones that have evolved from the same starting population but only differed in their resistance profile. DNA extractions were done on each isolate by MicrobesNG using in-house protocols (MicrobesNG Sequencing Service Methods, 2021). WGS was performed by MicrobesNG using an Illumina Novaseq 6000 to create 250 base pair (bp) paired-end reads. In-house processing by MicrobesNG trimmed the adapters and removed reads with a quality <15.

To further understand genetic changes that resulted in increased growth rates of phage-resistant *Variovorax*, cultures from week 7 monoculture lines of evolution were sent for population sequencing. Overnight cultures (120 µl frozen stock in 1/64 TSB) was grown shaking at 28°C. 1.8 ml was spun at 16 000 × *g* for 5 min (Progen GenFuge 24D centrifuge). The supernatant was removed and another 1.8 ml of fresh sample was added to each tube and centrifuged for a further 5 min and supernatant removed. The Qiagen DNeasy Ultra Clean DNA Extraction Kit reagents and protocol were used to extract the gDNA from the cultures. The concentration of gDNA in each sample was determined using the QuBit dsDNA HS Assay kit regents and protocol. Sequencing was done by the University of Liverpool’s Centre for Genomic Research (CGR) using an Illumina Novaseq to create 150 bp paired end reads. The CGR trimmed for the presence of Illumina adapter sequences using Cutadapt (v1.2.1) and removed reads with a minimum quality score of 20 and which were shorter 15 bp in length.

For both clonal and population sequencing, we further filtered the trimmed reads to remove Illumina adapter sequences and for reads with a quality score below 10 using TrimGalore (v0.6.4_dev). We used the breseq pipeline (v0.36)—a computational tool for analysing short-read DNA data—to map the trimmed reads to a high quality reference genome of *Variovorax* sp. (GenBank accession: GCA_037482385.1) and predict genetic variants (Deatherage and Barrick 2014). As our reference genome has two contigs, we added the parameters ‘-c’ and ‘—contig-reference’ so that all the sequences are assumed to have the same average coverage and coverage distribution. In the clonal sequencing, 97.6% of reads mapped to the reference genome (minimum of 95.4%), giving on average ∼876 989 reads per replicate (minimum 444 292) with an average coverage of 62.21 (minimum of 31.84). We only investigated genetic variants that were at a frequency of 1 (we do not expect polymorphisms in any of the predicted genetic variants). For the population sequencing, we ran breseq in polymorphism mode (-p). Variants with <5% frequency in the population samples were removed.

### Phage resistance and competition assay

We determined if phage resistance conferred a growth rate benefit to *Variovorax*, thereby leading to higher bacterial densities. Twelve clones per replicate from week 2 monoculture-lines were isolated and grown in 96-well plates with 150 µl TSB. Phage resistance was determined by streaking isolated colonies against the ancestral phage (streak assay). Week 2 isolates were chosen as this time-point preceded changes in bacterial density from phage exposure and phage extinction. We elected for phage-resistant clones generated during experimental evolution (instead of selecting spontaneous mutants) so that resistance mutations/mechanisms (and therefore phenotypic effects) were consistent with those from the evolution experiments. One phage-resistant clone from the phage resistance assays was chosen for each replicate (*n* = 6), and one clone from each no-phage treatment replicate was isolated as a phage-susceptible control (*n* = 6). Each colony was grown individually in 1/64 TSB individually as described previously. In addition, marked (ancestor-cp; chloramphenicol resistance; Sünderhauf et al. 2023) and unmarked ancestral *Variovorax* sp. were grown. Competition assays were used as a difference in growth rate between resistant to ancestral or susceptible strains will indicate a difference in growth rates and resource acquisition. Cultures were diluted to normalize densities as described previously. The unmarked ancestral strain and each phage-resistant and susceptible colony was competed against the marked ancestral strain at equal starting densities [∼3 × 10^6^ CFUs of each competitor inoculated (30 µl diluted culture)]. Diluted cultures used in inoculation were frozen and plated on KB agar as above for starting density estimation. Cultures were grown for 1 week as previous and plated from frozen onto KB and KB–chloramphenicol (25 µg/ml) agar. Relative fitness was calculated from the ratio of the estimated Malthusian parameters, *m*_resistant_, *m*_susceptible_, or *m*_ancestor_: *m*_ancestor-cp_, which were calculated as *m* = ln(*N*_1_/*N*_0_), where *N*_1_ is the final density and *N*_0_ is the starting density (Lenski et al. 1991).

### Growth curves

Next, we were interested if phage resistance had shifted the growth curve of *Variovorax* populations thereby leading to higher densities at transfer. 120 µl from each monoculture line from week 7 were revived in 6 ml growth medium and grown shaking for 2 days at 28°C. Densities were normalized as previous and 10 µl was inoculated into fresh growth medium. Every 24 h for 1 week, cultures were vortexed and optical density (OD_600_; Multiskan Sky) was measured from a 200 µl sample.

To characterize the log/exponential growth phase of phage-resistant and -susceptible cultures at a higher resolution, week 7 cultures were revived as previous and 20 µl of diluted culture (∼10^6^ CFUs) were inoculated into 180 µl growth medium. Optical density reads were measured every 45 min for 24 h.

### Supernatant assay

We hypothesized that higher densities and shifts in growth curve may be caused by production of less-toxic metabolites. This hypothesis was tested using a supernatant assay. Week 7 cultures were revived and normalized as previous using five replicates of resistant and susceptible populations (one replicate each of susceptible and resistant cultures became contaminated and were not used in the experiment). Two replicates of each week 7 culture (two technical replicates for each biological replicate) were established to ensure enough supernatant was available for the reciprocal transplant assay. Cultures were grown for 1 week. On day 5, fresh week 7 cultures were revived and grown for 2 days. Week-old cultures were sterilized by passing medium through a 0.22-µm filter syringe. Replicate supernatants from phage-resistant and -susceptible populations were pooled into separate supernatant stocks. To separate vials, 3 ml of phage-resistant and -susceptible supernatant was added to 25 ml vials. Two-day (fresh) culture densities were normalized and each culture was inoculated into both supernatant types. This established two controls in which cultures were grown in the same supernatant (e.g. phage resistant in phage-resistant supernatant) and treatments in which cultures were transplanted into the opposite supernatant (e.g. phage resistant in phage-susceptible supernatant and *vice versa*). Cultures were grown for 2 days and plated as previous.

### Phage resistance and community structure

Next, we assayed whether resistance to phage gives *Variovorax* an advantage over *Ochrobactrum* and *Pseudomonas* when phages are absent. Six independent phage-resistant clones and six independent phage-susceptible clones were isolated from phage and no phage week 2 monoculture evolution lines respectively. ‘No phage’ lines were selected for isolation of phage-susceptible clones as phage resistance was too high in ‘phage’ lines for enough individual susceptible clones to be isolated. Comparison between ‘no phage’ and ‘phage’ lines accounts for any general lab adaptation. Each clone and ancestral *Variovorax* were individually grown for 2 days at 28°C, shaking (180 rpm) in 6 ml 1/64 TSB. Ancestral *Pseudomonas* and *Ochrobactrum* isolates were also grown in the same conditions. Isolate densities were normalized to 10^5^ CFUs/ml and 10 µl added to relevant vials. Each *Variovorax* clone (ancestral, resistant, and susceptible) was grown as a polyculture with *Pseudomonas* and *Ochrobactrum*, with six replicates per treatment. Cultures were grown static for 1 week at 28°C, then frozen as previous and plated from frozen.

### Statistical analyses

All data were analysed using R (v.4.2.1) in RStudio (Team 2013) and all plots were made using the package ‘*ggplot2*’ (Wickham 2016). Model simplification was conducted using likelihood ratio tests and Tukey’s *post hoc* multiple comparison tests were done using the R package ‘*emmeans*’ (Lenth 2018).

*Variovorax* densities were analysed in a linear mixed effects model with density (log10 CFU/ml) tested against interacting fixed effects of phage exposure, culture type (polyculture or monoculture) and time (weeks 2, 4, 6, and 8) with a random effect of treatment replicate. Clonal densities (log10 CFU/ml) from clonal growth experiments were analysed against interacting fixed effects of phage exposure and time (week 2 or 4) with random effects of treatment replicate and block (experiment conducted in two blocks for feasibility). In competition assays, relative fitness was analysed in a linear model with a fixed effect of competitor (ancestor, phage resistant, or susceptible).

One week growth curves were analysed in a mixed effects model with optical density tested against a fixed term of day and interacting fixed effects of treatment (phage resistant/susceptible) and a quadratic term of day to account for the nonlinear relationship. A random effect of treatment replicate was included. 24-h assays of bacterial growth were used to estimate exponential growth rate. One replicate was removed from the no-phage treatment for failing to grow. Per replicate, exponential growth was estimated using rolling regression, taking the steepest slope of the linear regression between *OD*_600_ and time in hours in a shifting window of every nine time points (∼6.75 h). A linear model tested growth rate against treatment.

The supernatant assay was analysed in a linear mixed effects model with density (log10 CFU/ml) analysed against interacting fixed effects of supernatant type and original treatment with a random effect of treatment replicate.

To test whether consistent genetic differences occur within treatments, we performed nonmetric multidimensional scaling on the Euclidean distance matrix of SNPs/indels and their proportions in each population using ‘*metaMDS*’ in the R package ‘*vegan*’ (Oksanen et al. 2019). Nonmetric multidimensional scaling aims to collapse information from multiple dimensions (i.e. from different populations and multiple SNPs/indels per population) into just a few, allowing differences between samples to be visualized and interpreted. Permutational ANOVA tests were run using ‘*vegan:: adonis*’, with Euclidean distance as the response term and treatment as the predictor variable. Additionally, we analysed whether evolution with phage affected the genetic difference of each population (phage, no phage) from the ancestral population. The genetic distance from the ancestral population was calculated as the sum of the difference of the proportion of each SNP/indel in each population from the ancestral proportion. Genetic distance was then analysed in a linear model analysing distance against phage presence/absence.

To assess whether *Variovorax* phage resistance influences community structure in the absence of phage, we analysed the relative proportion of each species in a generalized linear mixed effects model. Here, each species proportion was analysed against interacting fixed effects of *Variovorax* phage resistance and species identity, with a random effect of treatment replicate and a binomial error structure.

## Results

### Phage-resistant clones have higher densities than phage-susceptible clones

In a previous study (Castledine et al. 2024a), we evolved *Variovorax* and its lytic phage in the presence and absence of two other bacteria–phage pairs (Fig. 1). We observed an interesting interaction with *Variovorax* densities increasing after 2 weeks of phage exposure (phage present: $\bar{x}\ $= 10^7.79^ CFU/ml, 95% CI = 10^7.74^–10^7.85^; phage absent: $\bar{x}\ $= 10^7.69^ CFU/ml, 95% CI = 10^7.63^–10^7.74^; ANOVA comparing models with and without phage: $\chi _1^2$= 7.21, *P* = .007; Tukey HSD: *P* = .013). This coincided with a fixation of phage resistance in polyculture (90.3%; SE ±6.6) and monoculture (100%), and extinction of phage after 2 weeks. As such, we investigated the hypothesis that phage resistance has led to increased *Variovorax* densities.

**Figure 2.**
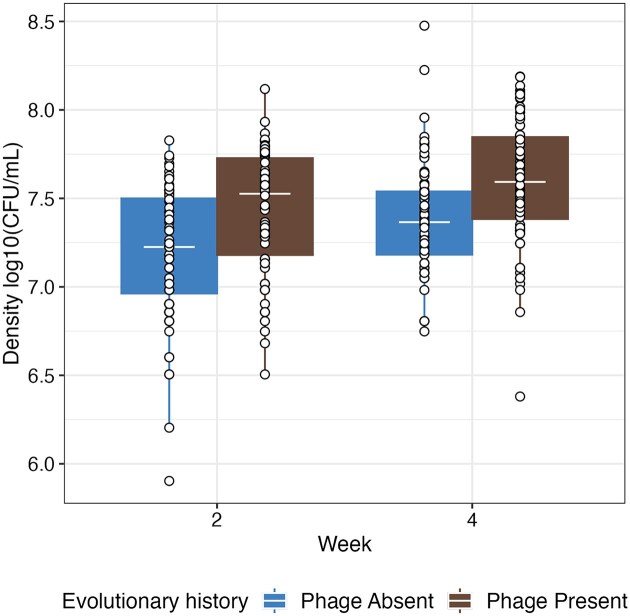
The density of individual clones (points), which have been grown after being isolated from evolution lines where phage have been absent or present. Tops and bottoms of the bars represent the 75th and 25th percentiles of the data, the middle lines are the medians, and the whiskers extend from their respective hinge to the smallest or largest value no further than 1.5× interquartile range. Points represent individual clones.

To assess whether higher densities in phage-exposed populations was likely a result of a genetic change (as opposed to epigenetic or physiological), we measured the densities of individual clones. Clones were isolated from monoculture treatment on weeks 2 (prephage loss) and 4 (postphage loss) and grown in isolation. No phage or other clones present so any density effects are purely heritable. All clones isolated from phage treatments were phage resistant as phage resistance reached fixation in monoculture, while all clones from the no-phage treatment were susceptible. Consistent with the population dynamics during experimental evolution, clones isolated from phage-exposed replicates had 66% higher densities ($\bar{x}\ $= 10^7.51^ CFU/ml, 95% CI = 10^5.60^–10^9.41^) than phage-unexposed clones ($\bar{x}\ $= 10^7.29^ CFU/ml, 95% CI = 10^5.38^–10^9.19^; ANOVA comparing models with and without phage: $\chi _1^2$= 6.60, *P* = .0102; Tukey HSD: *P* = .018; Fig. 2). Additionally, there was a significant independent effect of time with clones from week 4 ($\bar{x}\ $= 10^7.49^ CFU/ml, 95% CI = 10^5.30^–10^9.68^) reaching higher densities than clones from week 2 ($\bar{x}\ $= 10^7.31^ CFU/ml, 95% CI = 10^5.12^–10^9.49^; Tukey HSD: *P* < .001; Fig. 2). Overall, these results suggest effects imposed by phage were hereditary as phage did not need to be actively present for effects to be observed.

### Phage-resistant populations have distinct mutations

Given the consistent higher growth rate that phage exposed populations and phage-resistant clones had over phage absent populations and phage-susceptible clones, we looked at whether there were underlying genetic changes that could explain this. We sequenced three resistant and three sensitive clones coexisting within three polyculture treatment replicates, allowing for a direct comparison between different clones that evolved from the same starting population but differed in their resistance profile. Of the phage-resistant clones, two-thirds clones had a missense mutation at the same locus (gene ID: 01575) and one had a substitute mutation at a nearby locus (gene ID: 01584; 10.4k bp downstream; this was also the only mutation in this clone) that were not found in phage-susceptible isolates. No other mutations were found at the same loci across clones. Single point mutations are consistent with surface modification forms of resistance (Beckett and Williams 2013, van Houte et al. 2016). Generally, few mutations were found, with one resistant clone having four mutations while the other two had only one mutation each. Similarly, one phage-susceptible clone had two mutations while the other two had one mutation. 01584 sequence (mutation found in one-third resistant clones) had no comparable genes in BLAST, however its proximity to 01575 could imply a similar function (Demerec and Hartman 1959, Ballouz et al. 2010).

We conducted pool sequencing analysis of 7 weeks of evolution in monoculture (*Pseudomonas* and *Ochrobactrum* absent), with the aim of identifying further genetic changes linked to phage resistance and increased density. Five-sixths phage-resistant populations had at least one genetic variant at high frequency (>50%) that was not present in the phage-susceptible populations, compared to only two-sixths of the phage-susceptible populations, suggesting specific mutations were selected by phage (Fig. S1). Mutations in gene 01584 (found in one-third clones taken from polyculture) were also found in four-sixths replicates of monoculture populations suggesting similar mechanisms of resistance between poly- and monoculture. However, a lack of convergence suggests phage resistance may be maintained by several different mechanisms that have the same phenotypic effects (resistance and higher population density) (see Supplementary results for full analysis).

### Phage-resistant clones do not have a fitness advantage

**Figure 3.**
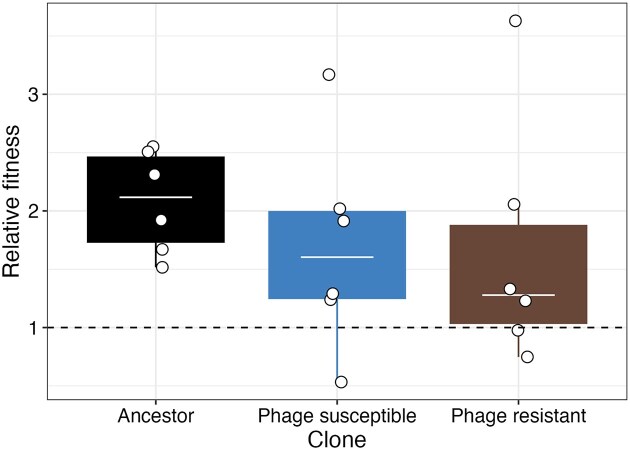
Relative fitness of ancestral, phage-susceptible, and -resistant isolates against a marked ancestral strain of *Variovorax*. Points represent independent replicates. Tops and bottoms of the bars represent the 75th and 25th percentiles of the data, the middle lines are the medians, and the whiskers extend from their respective hinge to the smallest or largest value no further than 1.5× interquartile range.

We next determined whether phage resistance conferred a fitness advantage in the absence of phage. In competition assays with a marked ancestral strain, relative fitness was not significantly affected by phage resistance (F_2,15_ = 0.460, *P* = .640; Fig. 3). The greater density associated with resistance did not therefore result in a competitive fitness advantage.

### Phage-resistant populations have greater persistence during the death phase

**Figure 4.**
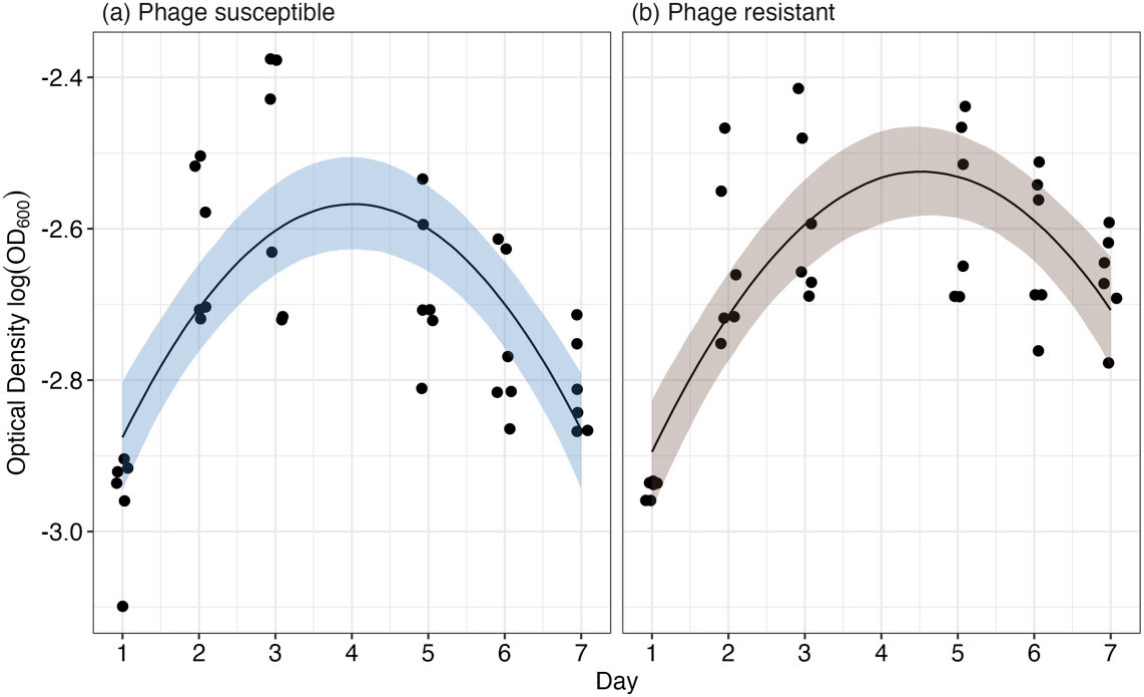
The 7-day growth curves of (a) phage-susceptible and (b) phage-resistant *Variovorax* populations that had evolved for 7 weeks. Points represent individual replicate populations measured repeatedly through time. Solid lines represent the mean predictions, and shaded bands represent the 95% confidence intervals of the best-fitted model.

**Figure 5.**
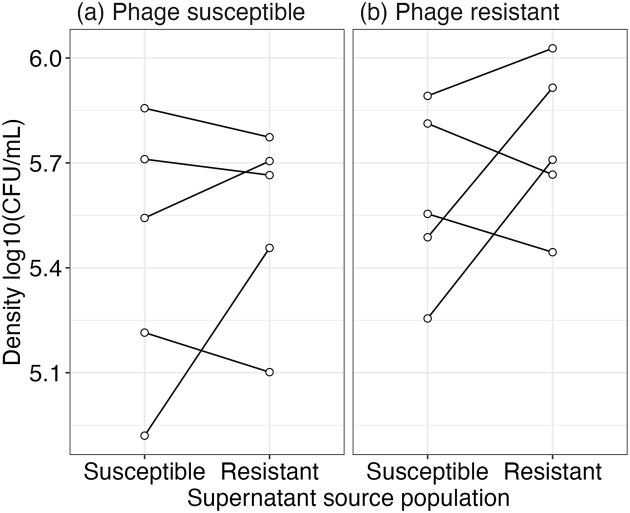
Density changes when populations, which are (a) phage susceptible and (b) phage resistant, are grown in supernatant isolated from either population. E.g. phage-susceptible growing in supernatant from phage-susceptible populations (same evolutionary history) or phage-susceptible growing in supernatant from phage-resistant populations (different evolutionary history). Points represent densities of individual replicates while lines indicate the change when that same population is grown in a different supernatant.

We next investigated if density benefits resulting from resistance arose from changes in growth dynamics. This may have arisen if, for example, logistic growth rates of resistant isolates were slowed, resulting in resources becoming less exhausted when cultures were transferred to fresh media on day 7. As such, we measured the growth curves of *Variovorax* populations isolated from week 7 of experimental evolution over 1 week. Here, there was a significant interaction between treatment and day of measurement (ANOVA comparing models with and without the interaction: $\chi _1^2$ = 7.25, *P* = .007), suggesting phage resistance had altered population growth dynamics (Fig. 4). Consistent with our previous observation based on CFU counts (Fig. 2), optical density after 1 week was higher in phage resistant ($\bar{x}\ $= 0.067, 95% CI = 0.062–0.072) than susceptible ($\bar{x}\ $= 0.057, 95% CI = 0.053–0.062) populations (Fig. 4). Consistent with an absence of fitness differences between susceptible and resistant isolates (Fig. 3), there was no significant difference in exponential growth rate measured in the 24 h growth rate assay (F_1,9_ = 0.622, *P* = .451) or carrying capacity in the 1 week growth rate assay (phage resistant: $\bar{x}\ $= 0.08, 95% CI = 0.075–0.085; phage susceptible: $\bar{x}\ $= 0.076, 95% CI = 0.072–0.081; Fig. 5). Phage-resistant isolates show similar growth dynamics to susceptible isolates, except they maintain higher densities during the death phase at day 7.

### The supernatant of phage-resistant populations is not less toxic

If bacterial populations can maintain a higher density during the stationary/death phase, this suggests that bacteria are potentially producing fewer toxic metabolites and/or are less susceptible to them. We investigated these possibilities by growing week 7 populations in spent supernatants from either their own treatment or the opposite treatment. However, population density was nonsignificantly different between populations irrespective of prior phage exposure ($\chi _1^2$ = 1.48, *P* = .224) or supernatant type ($\chi _1^2$ = 2.12, *P* = .145; interaction between effects: $\chi _1^2$ = 0.148, *P* = .7004; Fig. 5).

### Indirect benefit of phage resistance impacts community structure

**Figure 6.**
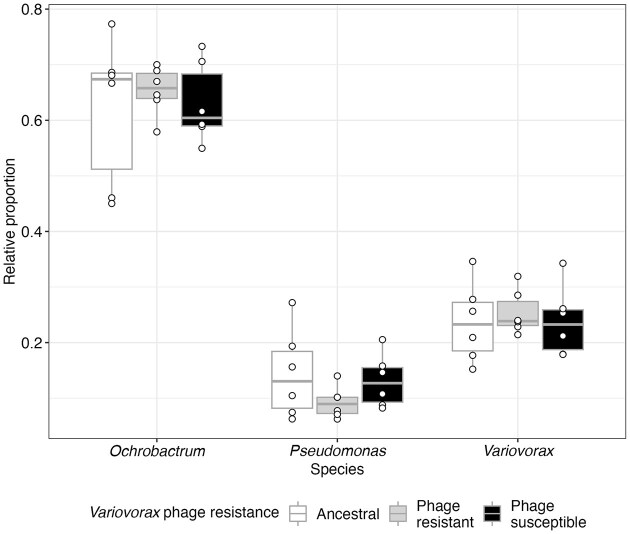
The relative proportion of each species in replicate communities when cultured with ancestral, phage-resistant, or -susceptible *Variovorax* isolates. Points represent individual treatment replicates. Tops and bottoms of the bars represent the 75th and 25th percentiles of the data, white lines indicate the medians, and whiskers extend from their respective hinge to the smallest or largest value no further than 1.5× the interquartile range.

Considering that phage resistance increased *Variovorax* densities, we considered whether this had implications for the structure of a simple microbial community. We had previously cultured *Variovorax* with two other species, *Ochrobactrum* and *Pseudomonas*, from which it derives a fitness benefit to the other species’ detriment (Castledine et al. 2024b). As such, we predicted that phage resistance increasing *Variovorax*’s density may have negative implications for the other two species. To this end, we reassembled communities with different *Variovorax* isolates with ancestral *Ochrobactrum* and *Pseudomonas*. Consistent with this prediction, the relative proportion of *Pseudomonas* in the community was significantly lower with phage-resistant *Variovorax* ($\bar{x}$ = 0.092, 95% CI = 0.08–0.11) compared to ancestral ($\bar{x}$ = 0.143, 95% CI = 0.128–0.158; Tukey HSD comparing *Pseudomonas* proportion with ancestral versus resistant *Variovorax*: estimate = 0.495, z-ratio = 5.02, *P* < .001) or phage-susceptible *Variovorax* ($\bar{x}$ = 0.128, 95% CI = 0.114–0.143; ANOVA comparing models with and without interaction between *Variovorax* phage resistance and species identity: $\chi _4^2$ = 34.1, *P* < .001; Tukey HSD comparing *Pseudomonas* proportion with resistant vs susceptible *Variovorax*: estimate = −0.367, z-ratio = −3.64, *P* < .001; Fig. 6). *Pseudomonas*’s relative proportion was nonsignificantly different when cultured with ancestral or phage-susceptible *Variovorax* (Tukey HSD: estimate = 0.129, z-ratio = 1.403, *P* = .339). Neither *Ochrobactrum*’s or *Variovorax’*s relative proportion was significantly affected by phage resistance in *Variovorax* (Tukey HSDs > 0.05, Table S1; Fig. 6).

## Discussion

Bacteriophages have been previously shown to positively influence bacteria densities, but only when propagated in monoculture and the mechanism was not determined (Gómez and Buckling 2011). Here, we showed that evolved resistance to phage changed the growth curves of *Variovorax* populations resulting in higher densities during the death phase. High levels of resistance, and the associated positive effect on density, was observed for several weeks past phage extinction within monocultures (no other bacterial species present) and in a community context. That this effect was consistent in both the presence and absence of other species is particularly interesting as the growth of this species is enhanced by the presence of the other two species (Castledine et al. 2020). As such, irrespective of the metabolic state the bacteria are in (feeding on the media or on supernatant), the phage had a positive effect on *Variovorax* density. This effect had consequences for community structure with phage resistance in *Variovorax* suppressing *Pseudomonas* in communities. These results demonstrate that phage resistance can give some species a relative advantage against others in community contexts, contrary to the prevailing wisdom of phage community ecology (Winter et al. 2010).

Phage resistance did not have a significant effect on exponential growth rate but did affect the death phase, with phage-resistant populations maintaining a higher density. Although we did not find evidence that this was linked to a change in metabolites produced after 1 week, it may reflect a broader ability of the bacteria being able to cope in stressful conditions. *Variovorax* is a metabolically plastic species and is capable of being lithoautotrophic or chemoorganotrophic, including the metabolism of toxic or complex compounds (Satola et al. 2013). Although a *Variovorax* phage was characterized recently, the authors did not examine resistance emergence (Decewicz et al. 2022), therefore little is known regarding the evolutionary interactions of this genus with phage.

A lack of convergent evolution meant we could not link genotype to phenotype in this study. Clones had few genetic changes, but fitness benefits from phage exposure were not linked to a single phage resistance mutation, although two-thirds clones had a missense mutation in the same protein. If this mutation and protein was the main mechanistic way phage-resistance increased fitness, we would expect it to also be present in the population sequencing, but no genetic changes in that locus were found. Instead, population sequencing revealed multiple mutations emerging and several reached high frequencies (>50%) in five-sixths phage treatment populations, although with a lack of convergent evolution. All high frequency genetic variants were in hypothetical proteins, making mechanistic inferences impossible within this project. Interestingly, only two no-phage culture had a unique mutation that reached high frequency (>50%) compared to five-sixths phage cultures, suggesting phage presence increased the likelihood of variants reaching a high frequency. Different mutations can confer the same phenotypic outcome, including phage resistance (Wright et al. 2018, 2019, Debray et al. 2022), which is likely to be the case in this study.

While resistance was clearly beneficial in the presence and absence of phage, reaching and being maintained at very high frequencies, we did not find any phage resistance emerge in phage-free controls across 8 weeks. This was presumably because there was no selective benefit of phage-resistant mutants competing against phage-sensitive clones in the absence of phage, and hence resistant mutations would not have increased in frequency. Nevertheless, we may expect this resistance-linked growth phenotype to be favoured in highly structured metapopulations, where limited dispersal can result in different patches seeded by single clones with the most productive patches (i.e. those seeded with resistant clones) producing more individuals to colonize new patches (Griffin et al. 2004). The resistance mutations may also allow populations to subsequently explore alternative evolutionary trajectories in the longer term (Salathé and Soyer 2008).

Importantly, phage resistance increasing *Variovorax* densities shifted community structure, resulting in lower proportions of *Pseudomonas. Variovorax* has been shown in previous work to benefit from the presence of *Pseudomonas* to *Pseudomonas*’ detriment (Castledine et al. 2024b). Increasing densities of phage-resistant *Variovorax*, therefore likely resulted in increased costs experienced by *Pseudomonas* in coculture. Phage lysis and phage resistance are typically assumed to be most costly to dominant community members (Winter et al. 2010). However, many examples exist of noncostly phage resistance and species dominating communities with high levels of phage resistance (Zhao et al. 2013, Castledine and Buckling 2024). Consequently, our results in-part support the ‘king-of-the-mountain’ hypothesis in which dominant community members are more able to evolve resistance owing to greater population sizes (Zhao et al. 2013, Giovannoni 2017)—albeit in this case likely by mutation than horizontal-gene-transfer (starting population isogenic). Synonymously, the ‘king-of-the-mountain’ hypothesis also relies on a low trade-off in defence and competition (Thingstad et al. 2014), evident in *Variovorax*.

Few examples exist of phages improving bacteria densities (Gómez and Buckling 2011), suggesting this observation may not be ubiquitous. However, much research has heavily focused on model strains of bacteria and phage, which are unlikely to be generalizable to all bacteria and phages (Castledine and Buckling 2024, Castledine et al. 2024a). Furthermore, different mechanisms of phage resistance have the potential to improve bacteria densities in specific contexts. For example, phages can select for increased biofilm production (Scanlan and Buckling 2012, Castledine et al. 2022) and persister populations (Fernández-García et al. 2023), which can further confer resistance to stressors such as antibiotics and immune cells (Crabbé et al. 2019, Niu et al. 2024). If phages can improve the density of soil bacteria, such as by driving host diversification (Brockhurst et al. 2004), this may have positive impacts on carbon cycling and crop yield (Dy et al. 2018). *Variovorax* species, for instance, are symbionts to plants and are found in the rhizosphere, where they can improve plant growth (Satola et al. 2013). Alternatively, if phages are used therapeutically (Oechslin 2018, Abedon 2019) then density enhancement of target bacterial pathogens is clearly detrimental. Work considering wider bacteria and phage pairs, beyond model systems, will allow greater generalizations to be made and understanding of how phages can affect their hosts in the short and long-term.

The influence of phages on bacterial populations are highly diverse, making predictions and generalizations challenging from model systems to phages in nature. Our work highlights the surprising outcomes that can arise from bacteria–phage interactions, and that the simple assumption that lytic phages negatively affect host densities may not always be true. Wider research analysing the effects of different phages on host bacteria, both during and after phage extinction would give important insight into how phage therapy may affect pathogens during infection and how phages operate in nature.

## Supplementary Material

fiaf027_Supplemental_Files

## Data Availability

All data and R code used in the analysis are available on GitHub (https://github.com/mcastledine96/Variovorax_phage_resistance_increases_density_2024). Assemblies and sequencing data are available from ENA (Project Accession PRJEB74093).

## References

[bib1] Abedon ST . Use of phage therapy to treat long-standing, persistent, or chronic bacterial infections. Adv Drug Deliv Rev. 2019;145:18–39. 31708017 10.1016/j.addr.2018.06.018

[bib2] Abedon ST . Ecology and evolutionary biology of hindering phage therapy: the phage tolerance vs. phage resistance of bacterial biofilms. Antibiotics. 2023;12:245. 36830158 10.3390/antibiotics12020245 PMC9952518

[bib3] Ballouz S, Francis AR, Lan R et al. Conditions for the evolution of gene clusters in bacterial genomes. PLoS Comput Biol. 2010;6:e1000672. 20168992 10.1371/journal.pcbi.1000672 PMC2820515

[bib4] Beckett SJ, Williams HTP. Coevolutionary diversification creates nested-modular structure in phage–bacteria interaction networks. Interface Focus. 2013;3:20130033. 24516719 10.1098/rsfs.2013.0033 PMC3915849

[bib5] Breitbart M, Bonnain C, Malki K et al. Phage puppet masters of the marine microbial realm. Nat Microbiol. 2018;3:754–66. 29867096 10.1038/s41564-018-0166-y

[bib6] Breitbart M . Marine viruses: truth or dare. Annu Rev Mar Sci. 2012;4:425–48. 10.1146/annurev-marine-120709-142805 22457982

[bib7] Brockhurst MA, Rainey PB, Buckling A. The effect of spatial heterogeneity and parasites on the evolution of host diversity. Proc R Soc Lond B. 2004;271:107–11. 10.1098/rspb.2003.2556 PMC1691556 15002778

[bib8] Buckling A, Wei Y, Massey RC et al. Antagonistic coevolution with parasites increases the cost of host deleterious mutations. Proc R Soc B. 2006;273:45–9. 10.1098/rspb.2005.3279 PMC1560003 16519233

[bib9] Castledine M, Buckling A. Critically evaluating the relative importance of phage in shaping microbial community composition. Trends Microbiol. 2024;32:957–69. 38604881 10.1016/j.tim.2024.02.014

[bib10] Castledine M, Padfield D, Buckling A. Experimental (co)evolution in a multi-species microbial community results in local maladaptation. Ecol Lett. 2020;23:1673–81. 32893477 10.1111/ele.13599

[bib12] Castledine M, Padfield D, Sierocinski P et al. Parallel evolution of *Pseudomonas aeruginosa* phage resistance and virulence loss in response to phage treatment *in vivo* and *in vitro*. eLife. 2022;11:e73679. 35188102 10.7554/eLife.73679 PMC8912922

[bib11] Castledine M, Padfield D, Schoeman M et al. Bacteria-phage (co)evolution is constrained in a synthetic community across multiple bacteria-phage pairs. bioRxiv. 2024a. 10.1101/2024.10.14.618343. PMC12178565 40536890

[bib13] Castledine M, Pennycook J, Newbury A et al. Characterizing a stable five-species microbial community for use in experimental evolution and ecology. Microbiology. 2024b;170. 10.1099/mic.0.001489. PMC11412253 39297874

[bib14] Chan BK, Sistrom M, Wertz JE et al. Phage selection restores antibiotic sensitivity in MDR *Pseudomonas aeruginosa*. Sci Rep. 2016;6:26717. 27225966 10.1038/srep26717 PMC4880932

[bib15] Clokie MRJ, Millard AD, Letarov AV et al. Phages in nature. Bacteriophage. 2011;1:31–45. 21687533 10.4161/bact.1.1.14942 PMC3109452

[bib16] Crabbé A, Jensen PØ, Bjarnsholt T et al. Antimicrobial tolerance and metabolic adaptations in microbial biofilms. Trends Microbiol. 2019;27:850–63. 31178124 10.1016/j.tim.2019.05.003

[bib17] Deatherage DE, Barrick JE. Identification of mutations in laboratory-evolved microbes from next-generation sequencing data using breseq. Methods Mol Biol. 2014;1151:165–88. 24838886 10.1007/978-1-4939-0554-6_12 PMC4239701

[bib18] Debray R, De Luna N, Koskella B. Historical Contingency Drives Compensatory Evolution and Rare Reversal of Phage Resistance. Mol Biol Evol. 2022;39:msac182. 35994371 10.1093/molbev/msac182 PMC9447851

[bib19] Decewicz P, Kitowicz M, Radlinska M. Characteristics and comparative genomic analysis of a novel virus, VarioGold, the first bacteriophage of Variovorax. Int J Mol Sci. 2022;23:13539. 36362326 10.3390/ijms232113539 PMC9658991

[bib20] Demerec M, Hartman PE. Complex loci in microorganisms. Annu Rev Microbiol. 1959;13:377–406.

[bib21] Dy RL, Rigano LA, Fineran PC. Phage-based biocontrol strategies and their application in agriculture and aquaculture. Biochem Soc Trans. 2018;46:1605–13. 30514766 10.1042/BST20180178

[bib22] Fernández-García L, Kirigo J, Huelgas-Méndez D et al. Phages produce persisters. Microb Biotechnol. 2023;17:e14543. 10.1101/2023.10.17.562728. PMC11297538 39096350

[bib23] Flynn KJ, Mitra A, Wilson WH et al. ‘Boom-and-busted’ dynamics of phytoplankton–virus interactions explain the paradox of the plankton. New Phytol. 2022;234:990–1002. 35179778 10.1111/nph.18042 PMC9313554

[bib24] Gandon S, Buckling A, Decaestecker E et al. Host-parasite coevolution and patterns of adaptation across time and space. J Evol Biol. 2008;21:1861–6. 18717749 10.1111/j.1420-9101.2008.01598.x

[bib25] Giovannoni SJ . SAR11 bacteria: the most abundant plankton in the oceans. Annu Rev Mar Sci. 2017;9:231–55. 10.1146/annurev-marine-010814-015934 27687974

[bib26] Gómez P, Buckling A. Bacteria-phage antagonistic coevolution in soil. Science. 2011;332:106–9. 21454789 10.1126/science.1198767

[bib27] Griffin AS, West SA, Buckling A. Cooperation and competition in pathogenic bacteria. Nature. 2004;430:1024–7. 15329720 10.1038/nature02744

[bib28] Harper D, Parracho H, Walker J et al. Bacteriophages and biofilms. Antibiotics. 2014;3:270–84.

[bib29] Jayaraman R . Hypermutation and stress adaptation in bacteria. J Genet. 2011;90:383–91. 21869495 10.1007/s12041-011-0086-6

[bib30] Koskella B, Lin DM, Buckling A et al. The costs of evolving resistance in heterogeneous parasite environments. Proc R Soc B. 2012;279:1896–903. 10.1098/rspb.2011.2259 PMC3311890 22171085

[bib31] Lennon JT, Khatana SAM, Marston MF et al. Is there a cost of virus resistance in marine cyanobacteria?. ISME J. 2007;1:300–12. 18043641 10.1038/ismej.2007.37

[bib32] Lenski RE, Rose MR, Simpson SC et al. Long-term experimental evolution in *Escherichia coli*. I. Adaptation and divergence during 2000 generations. Am Nat. 1991;138:1315–41.

[bib33] Lenth R . Emmeans: Estimated Marginal Means, Aka Least-squares Means. R Package Version. CRAN, 2018.

[bib34] Li N, Zeng Y, Wang M et al. Characterization of phage resistance and their impacts on bacterial fitness in *Pseudomonas aeruginosa*. Microbiol Spectr. 2022;10:e02072–22. 10.1128/spectrum.02072-22 PMC9603268 36129287

[bib35] Liao H, Liu C, Ai C et al. Mesophilic and thermophilic viruses are associated with nutrient cycling during hyperthermophilic composting. ISME J. 2023;17:916–30. 37031344 10.1038/s41396-023-01404-1 PMC10202948

[bib36] Niu H, Gu J, Zhang Y. Bacterial persisters: molecular mechanisms and therapeutic development. Sig Transduct Target Ther. 2024;9:174. 10.1038/s41392-024-01866-5 PMC11252167 39013893

[bib37] Oechslin F . Resistance development to bacteriophages occurring during bacteriophage therapy. Viruses. 2018;10:351. 29966329 10.3390/v10070351 PMC6070868

[bib38] Oksanen J, Simpson GL, Blanchet FG et al. vegan: Community Ecology Package. CRAN, 2019.

[bib39] Oliveira NM, Martinez-Garcia E, Xavier J et al. Biofilm formation as a response to ecological competition. PLoS Biol. 2015;13:e1002191. 26158271 10.1371/journal.pbio.1002191 PMC4497666

[bib40] Pal C, Maciá MD, Oliver A et al. Coevolution with viruses drives the evolution of bacterial mutation rates. Nature. 2007;450:1079–81. 18059461 10.1038/nature06350

[bib41] Payne LJ, Meaden S, Mestre MR et al. PADLOC: a web server for the identification of antiviral defence systems in microbial genomes. Nucleic Acids Res. 2022;50:W541–50. 35639517 10.1093/nar/gkac400 PMC9252829

[bib42] Salathé M, Soyer OS. Parasites lead to evolution of robustness against gene loss in host signaling networks. Mol Syst Biol. 2008;4:202. 18628743 10.1038/msb.2008.44 PMC2516366

[bib43] Satola B, Wübbeler JH, Steinbüchel A. Metabolic characteristics of the species *Variovorax paradoxus*. Appl Microbiol Biotechnol. 2013;97:541–60. 23192768 10.1007/s00253-012-4585-z

[bib44] Scanlan PD, Buckling A. Co-evolution with lytic phage selects for the mucoid phenotype of *Pseudomonas fluorescens* SBW25. ISME J. 2012;6:1148–58. 22189495 10.1038/ismej.2011.174 PMC3358020

[bib45] Sünderhauf D, Klümper U, Gaze WH et al. Interspecific competition can drive plasmid loss from a focal species in a microbial community. ISME J. 2023;17:1765–73. 37558861 10.1038/s41396-023-01487-w PMC10504238

[bib46] The R Core Team . R: A Language and Environment for Statistical Computing. Vienna: The R Foundation, 2013.

[bib47] Thingstad TF, Våge S, Storesund JE et al. A theoretical analysis of how strain-specific viruses can control microbial species diversity. Proc Natl Acad Sci USA. 2014;111:7813–8. 24825894 10.1073/pnas.1400909111 PMC4040589

[bib48] van Houte S, Buckling A, Westra ER. Evolutionary ecology of prokaryotic immune mechanisms. Microbiol Mol Biol Rev. 2016;80:745–63. 27412881 10.1128/MMBR.00011-16 PMC4981670

[bib49] Wickham H . Ggplot2: Elegant Graphics for Data Analysis. New York, NY: Springer, 2016.

[bib50] Winter C, Bouvier T, Weinbauer MG et al. Trade-offs between competition and defense specialists among unicellular planktonic organisms: the “Killing the Winner” hypothesis revisited. Microbiol Mol Biol Rev. 2010;74:42–57. 20197498 10.1128/MMBR.00034-09 PMC2832346

[bib51] Wright RCT, Friman V-P, Smith MCM et al. Cross-resistance is modular in bacteria–phage interactions. PLoS Biol. 2018;16:e2006057. 30281587 10.1371/journal.pbio.2006057 PMC6188897

[bib52] Wright RCT, Friman V-P, Smith MCM et al. Resistance evolution against phage combinations depends on the timing and order of exposure. mBio. 2019;10:e01652–19. /mbio/10/5/mBio.01652-19.atom. 31551330 10.1128/mBio.01652-19 PMC6759759

[bib53] Zhao Y, Temperton B, Thrash JC et al. Abundant SAR11 viruses in the ocean. Nature. 2013;494:357–60. 23407494 10.1038/nature11921

