# Supplementary material for: Microbial community structure is affected by phage-resistance associated increases in host density
Source: FEMS Microbiol Ecol. 2025 Mar 18;101(4):fiaf027. doi: 10.1093/femsec/fiaf027 (PMC11953036; doi:10.1093/femsec/fiaf027)

**Supplementary Information**

**Supplementary results: pool sequencing**

In total, we found 470 genetic variants, with 239 shared between resistant and susceptible populations. Phage resistant populations had 117 unique variants (within 60 genes) while phage susceptible populations had 114 unique variants (within 64 genes). Most variants were found at low frequency (96.8% of variants at <50% frequency) with resistant and susceptible populations having on average 147 variants. 5/6 phage resistant populations had at least one unique genetic variant at high frequency (>50%) compared to only 2/6 of the phage susceptible populations, suggesting specific mutations were selected by phage.

While we did not observe mutations in 01575 (mutation found in 2/3 resistant clones), mutations were found in 01584 (mutation found in 1/3 resistant clones) in 4/6 phage resistant populations (92.7%, 68.2%, 12.3%, 9.6%). Mutations selected to high frequency were also found in *prpR* (69.3% in 1 population; transcription regulator gene) and in five other hypothetical proteins or intergenic regions (Figure S1). The lack of convergence suggests phage resistance may be maintained by several different mechanisms that have the same phenotypic effects (resistance and higher population density). As a result, we did not find phage presence significantly differed in genetic composition (PERMANOVA: F_1,10_ = 0.889, p = 0.81, Figure S2) or distance from the ancestral genome (ANOVA: F_1,10_ = 0.547, p = 0.477).

**
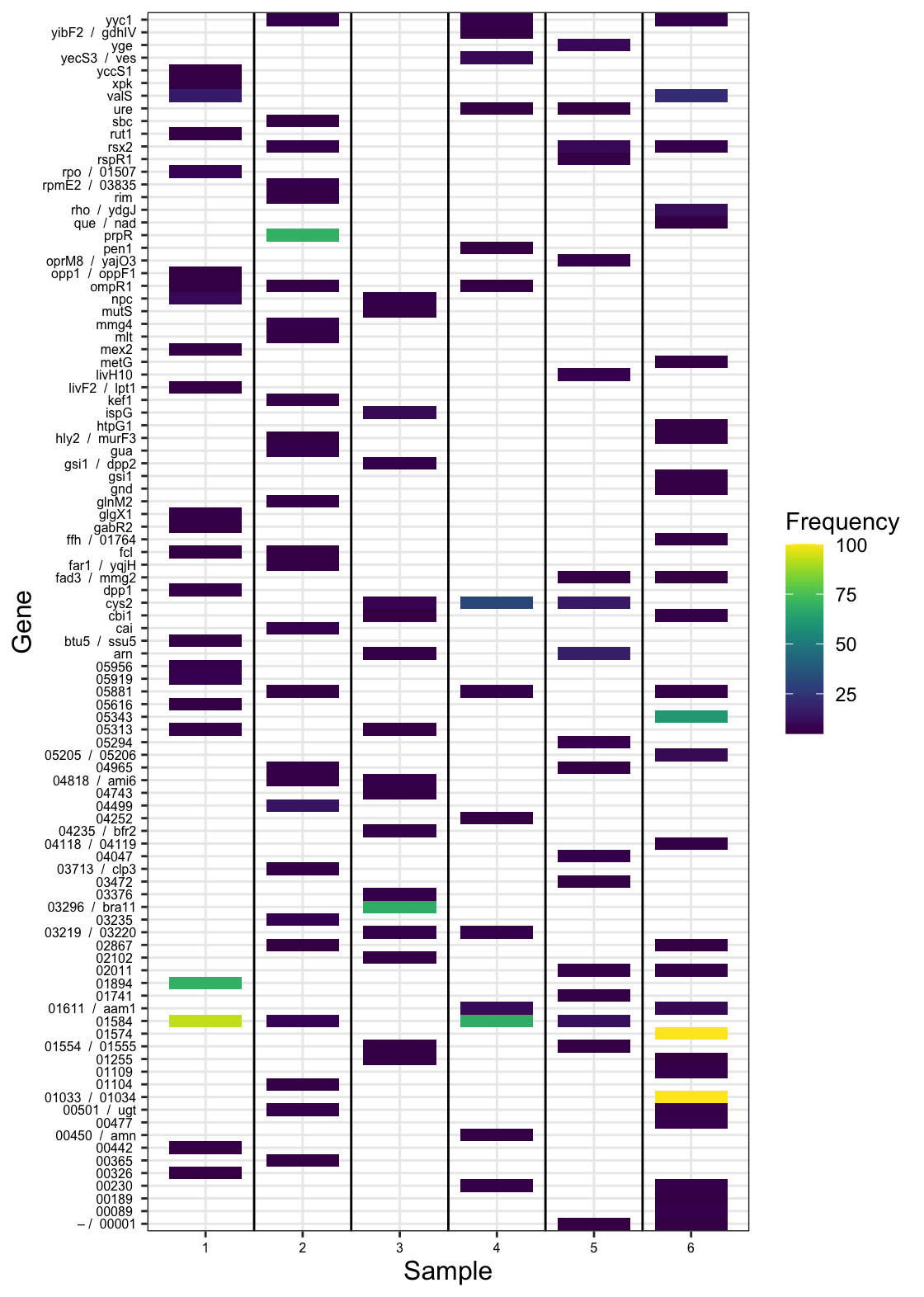
**

Figure S1. The frequency of genetic variants found in phage resistant but not phage susceptible populations. Where multiple variants are found in the same gene, the average has been presented. Each replicate population is identified on the x-axis. Genes of known function are named on the y-axis while hypothetical proteins are given a numerical denomination. Gene names separated by a forward slash indicate mutations in intergenic regions. Samples indicate each of the six replicate populations that evolved in the presence of phage.


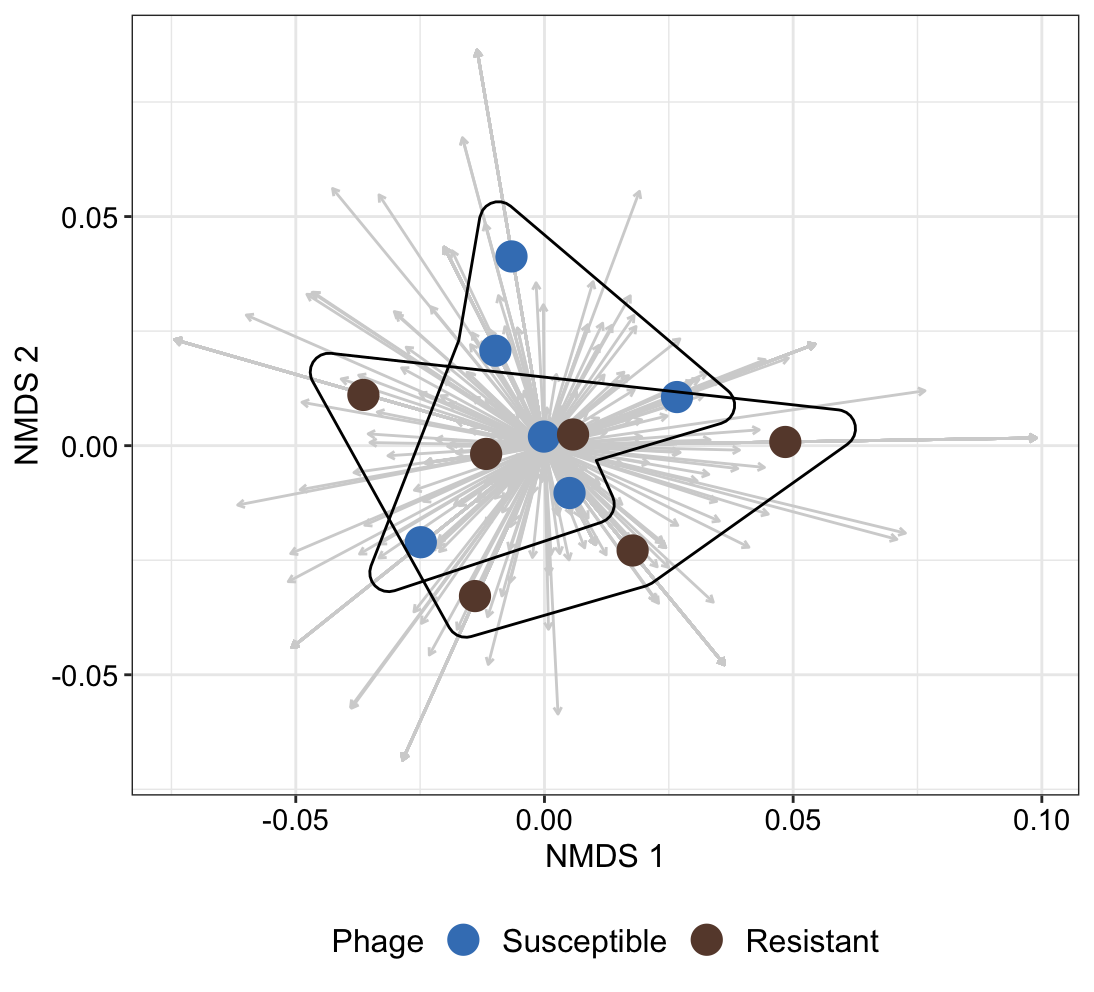


Figure S2. The divergence of each treatment group as indicated by Euclidean distances of each gene (arrows) from the ancestral genome. Points represent individual replicates sequenced from phage resistant or susceptible treatment groups.

**Supplementary Table**

Table S1. Tukey HSD comparisons comparing the relative proportion of *Ochrobactrum* (O), *Pseudomonas* (P) and *Variovorax* (V) when the *Variovorax* strain is the ancestral type, phage resistant or susceptible. P-values are adjusted by the tukey method for comparing a family of three estimates.


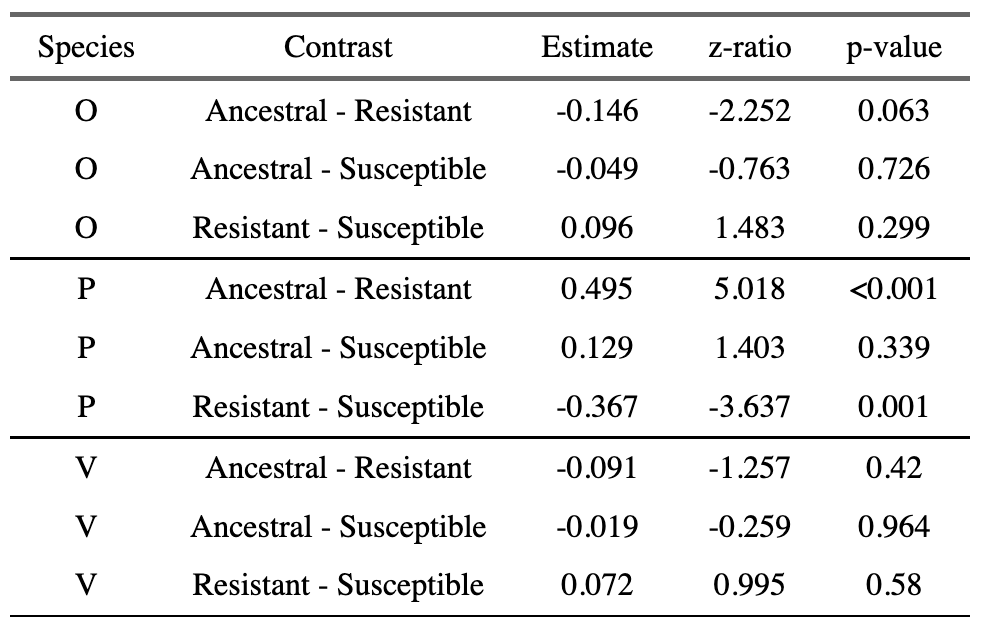

Supplement: fiaf027_Supplemental_Files [file fiaf027_supplemental_files.zip › Supplementary Information_femsv.docx]
